# Association of polymorphic variants in GEMIN genes with the risk of depression in a Polish population

**DOI:** 10.7717/peerj.14317

**Published:** 2022-11-15

**Authors:** Mateusz Kowalczyk, Edward Kowalczyk, Monika Gogolewska, Maciej Skrzypek, Monika Talarowska, Ireneusz Majsterek, Tomasz Poplawski, Paweł Kwiatkowski, Monika Sienkiewicz

**Affiliations:** 1 Babinski Memorial Hospital, Lodz, Poland; 2 Department of Pharmacology and Toxicology, Medical University of Lodz, Lodz, Poland; 3 Department of Clinical Chemistry and Biochemistry, Medical University of Lodz, Lodz, Poland; 4 Department of Clinical Psychology and Psychopathology, University of Lodz, Lodz, Poland; 5 Department of Microbiology and Pharmaceutical Biochemistry, Medical University of Lodz, Lodz, Poland; 6 Department of Diagnostic Immunology, Pomeranian Medical University in Szczecin, Szczecin, Poland; 7 Department of Pharmaceutical Microbiology and Microbiological Diagnostic, Medical University of Lodz, Lodz, Poland

**Keywords:** GEMIN, Polymorphism, Depression

## Abstract

**Background:**

The role of miRNA in depression is widely described by many researchers. miRNA is a final product of many genes involved in its formation (maturation). One of the final steps in the formation of miRNAs is the formation of the RISC complex, called the RNA-induced silencing complex, which includes, among others, GEMIN proteins. Single-nucleotide polymorphisms (SNPs) may lead to disturbance of miRNA biogenesis and function. The objective of our research was to assess the relationship between the appearance of depression and single nucleotide polymorphisms in the GEMIN3 (rs197388) and GEMIN4 (rs7813; rs3744741) genes. Our research provides new knowledge on the genetic factors that influence the risk of depression. They can be used as an element of diagnostics helpful in identifying people at increased risk, as well as indicating people not at risk of depression.

**Methods:**

A total of 218 participants were examined, including individuals with depressive disorders (*n* = 102; study group) and healthy people (*n* = 116, control group). All the patients in the study group and the people in the control group were non-related native Caucasian Poles from central Poland. Blood was collected from study and control groups in order to assess the SNPs of GEMIN genes.

**Results:**

An analysis of the results obtained showed that in patient population, the risk of depression is almost doubled by polymorphic variants of the genes: rs197388/GEMIN3 genotype A/A in the recessive model and rs3744741/GEMIN4 genotype T/T, codominant and recessive model. The dual role of rs7813/GEMIN4 is noteworthy, where the G/A genotype in the codominant and over dominant model protects against depression.

## Introduction

Before the COVID-19 pandemic, approximately 350 million people worldwide suffered from depression, according to estimates by the World Health Organization (GBD: 2017 Disease and Injury Incidence and Prevalence Collaborators, 2018). The worldwide distribution of the so-called major depression (MD), including major depressive disorder (MDD), was estimated to range from 3% in Japan to 16.9% in the US (in most countries, the prevalence ranged from 8% to 12%) (Arias-de la Torre et al., 2021).

MDD is a chronic and debilitating disease (Otte et al., 2016). It is defined as a complex and heterogeneous syndrome that comprises a wide spectrum of symptoms, including anhedonia, sleep disorders, decreased appetite and energy, depressed mood, decreased concentration, and suicidal thoughts (Pitsillou et al., 2020). Despite the clinical heterogeneity, MDD patients are still grouped according to one diagnostic unit (World Health Organization, 2004). The etiology and pathophysiology of MDD is not well understood due to its complex endophenotype, but recent research suggests that MDD is the result of complex genetic and environmental interactions. However, it remains unclear how the above factors impair brain function, leading to the onset of depression and persistent behavioral changes. Recent studies suggest that epigenetic factors provide a bridge between the environment and genetics; therefore, epigenetic research will allow the development of individualized MDD therapies, the classification of MDD subtypes and hereby increase the effectiveness of existing therapies (Park et al., 2019).

There are some major epigenetic mechanisms that have been extensively studied in MDD. DNA methylation, histone modifications, chromosome remodeling, and RNA regulation by noncoding RNAs such as microRNAs (miRNAs) and long-non-coding RNAs (lncRNAs) were analyzed (Penner-Goeke & Binder, 2019; Aristizabal et al., 2020). Of these, studies focusing on miRNAs are at the forefront. Yuan et al. (2018) evaluated miRNAs in peripheral tissues of MDD patients. They noticed changes in 178 different miRNAs compared to the control group. Only miRNA-132 has been replicated in four independent studies. However, it should be emphasized that miRNAs are a final product of many genes (Laffont & Rayner, 2017; Ambros & Ruvkun, 2018). One of the last steps in the origination of miRNAs is the formation of the RISC complex, known as the RNA-induced silencing complex. RISC is considered to be an effector mechanism in the RNA-mediated gene silencing pathway (Pratt & MacRae, 2009). The miRNAs in this complex combine, among others, with proteins of the ARGONAUTE family (AGO) and proteins GEMIN3 and GEMIN4 (Curmi & Cauchi, 2018; Wu et al., 2020).

Allelic heterogeneity of genes encoding AGO and GEMIN proteins lead to a disruption of miRNA biogenesis and function (Fang et al., 2016; Nowak & Sarshad, 2021). Our attention was drawn to the work of He et al. (2012) who sought a relationship between single nucleotide polymorphisms (SNPs) in AGO and GEMIN genes and a risk of depression. They discovered that AGO1 rs636832 was significantly associated with depression and that GEMIN4 rs7813 did not affect susceptibility to depression. These observations suggest that SNPs in miRNA processing genes may influence the risk of depression. Therefore, we decided to check whether the polymorphism of the GEMIN3 and GEMIN4 genes is associated with the risk of depression in the Polish population (Trivedi, 2020).

The aim of our research was to assess a relationship between the appearance of depression and single nucleotide polymorphisms (SNP) in genes. GEMIN3 (rs197388), GEMIN4 (rs7813; rs3744741). Our data can contribute to enriching knowledge about genetic factors influencing the risk of depression, as well as serve as a diagnostic marker in identifying people at increased risk of depression, and indicate those without such a risk introduction should briefly place the study in a broad context and highlight why it is important.

## Materials & Methods

### Subjects

The research was conducted in the period from January 2019 to December 2020. A total of 218 people were examined. All patients in the study group and the control group were native, unrelated native Caucasian Poles from central Poland. Blood was drawn from participants (the study and control groups) for the purpose of assessing the GEMIN gene polymorphism. In both groups, people with chronic inflammatory diseases, with neurological disorders, with organic disorders, under oncological treatment, metabolically incompetent, with injuries (including head injuries), with autoimmune diseases, addicted to psychotropic drugs and narcotics, and those who refused to sign an informed consent were excluded from the study. None of the participants had cancer. Each of the respondents expressed their consent in writing to participate in the study according to the protocol approved by the Bioethics Committee of the Medical University of Lodz no. RNN/402/18 of the European Commission of 10 December 2018. Each person in the study and control groups also completed the Hamilton Depression Scale form, which allowed controls with abnormal results.

The study group consisted of 102 people with MDD diagnosed with a depressive episode or the presence of recurrent MDD hospitalized in the Department of Adult Psychiatry, Medical University of Lodz and in the Specialist Psychiatric Health Care Complex in Lodz, Hospital of J. Babiński. The patients were enrolled in the study group based on diagnostic criteria included in ICD-10 (International Statistical Classification of Diseases and Related Health Problems) for depressive episodes (F32) and recurrent MDD (F33.0–F33.8). All patients were examined upon admission to treatment. The study group included psychiatric patients hospitalized for the first time and not previously treated for MDD, as well as people who had been treated pharmacologically for many years, admitted to the ward to receive a modified therapy, or due to deterioration of health (another affective episode).

The control group consisted of 116 healthy people with a negative family history of mental illness and a score of 0 to 7 points on the Hamilton Depression Scale. Exclusion criteria, as in the study group, were neurological, neoplastic, and inflammatory diseases.

### DNA Isolation

DNA for genotyping was isolated from blood. Blood samples were collected in anticoagulant EDTA tubes (Sarstedt, Nümbrecht, Germany). The QIAamp DNA Blood Mini Kit (Qiagen, Chatsworth, CA, USA) was used for DNA isolation according to the protocol provided with the kit by the manufacturer. After isolation, DNA samples were stored at −20 °C in TE buffer (pH 8.0). The DNA concentration and purity of the DNA preparations were determined using the spectrophotometric method by measuring absorbance at 260 and 280 nm on a nanodrop microspectrofluorimeter (Thermo Scientific, Waltham, MA, US).

### Determination of SNP

The frequency of polymorphic variants of genes: GEMIN3 (rs197388), GEMIN4 (rs7813; rs3744741), was determined using TaqMan® SNP Genotyping Assays and TaqMan Universal PCR Master Mix, no AmpErase UNG kits (Applied Biosystems, Foster City, CA, US). The kit was supplied with primers and fluorescently labeled molecular probes, which allow genotype reading during real-time DNA polymerase chain reaction analysis. The markings were made according to the recommendations attached by the manufacturer. The reaction was carried out using the Stratagene Mx3005p system (Agilent Technologies, Santa Clara, CA, USA).

### Statistical analysis

Allelic and haplotype frequencies as well as Hardy-Weinberg Equilibrium (HWE) were calculated with SNPstats (Solé et al., 2006). This tool was also used to assess the contribution of studied polymorphisms to the risk of depression in multiple genetic models. In genetic association studies, it is common practice to assume an additive model (Gaye & Davis, 2017) and the genetic model should be matched to the disease inheritance pattern. Since the pattern of inheritance of depression is not determined yet, we decided to present results for four models: co-dominant, dominant, recessive, and over-dominant. Odds ratios (ORs) and 95% confidence intervals (CIs) were estimated using a logistic regression model adjusted by age and gender. We also calculated the statistical power with Genetic Power Calculator (Purcell, Cherny & Sham, 2003).

## Results

### Characteristics of the study and control groups

The age of all participants (*n* = 218) was 40 ± 14 (ranged from 19 to 81). In the study (*n* = 102) and control (*n* = 116) groups, the age was 44 (ranged from 19 to 81) and 33 (ranged from 21 to 60), respectively. Women prevailed (more than 70% of participants in the control group and 80% in the group with recurrent MDD) both in people with recurrent MDD and in the control group. The characteristics of the study and control groups regarding gender and age are presented in Table 1.

### Intensity of depression symptoms according to the HDRS scale

Of the study group patients with MDD according to the HDRS (Hamilton Depression Scale), 3% demonstrated very severe disorders, over 63% severe, 21%—moderate and 11% mild. The intensity of depression symptoms in the study group is shown in Table 2.

### The Hardy Weinberg Equilibrium analysis

The results of the statistical analysis of deviations from the Hardy-Weinberg equilibrium (HWE) law are presented in Table 3. The genotype distribution for the rs7813 polymorphism was consistent with the Hardy-Weinberg equilibrium law. The frequency of the distributions of the remaining analyzed polymorphisms showed a deviation from the HWE law (*p* < 0.05).

### Analysis of the relationship between the occurrence of depression and the polymorphic variants of GEMIN genes

This evaluation was carried out using association studies. These are population studies that determine whether a specific allele of a particular gene is more common in a group of unrelated affected individuals than in healthy individuals. For each polymorphism, the frequency of individual genotypes is presented in relation to the presence or absence of depression. Four genetic models were analyzed: codominant, dominant, recessive and over-dominant. The codominant model is the most general model which assumes that each genotype generates a distinct, independent risk. This model compares genotypes that are heterozygous and homozygous for the variant allele with those homozygous for the most common allele. The dominant and recessive model assumes that the variant allele is sufficient to increase the risk of depression (one for the dominant model and two for the recessive model). The last model, a over-dominant one, assumes that only heterozygote contributes to the risk of depression. Table 4 shows the distribution of genotypes of the rs197388/GEMIN3 polymorphism compared to depression. A relationship between the A/A genotype in the recessive model and depression has been demonstrated. The above genotype increased the risk of developing depression (OR = 1.96).

**Table 1:** Comparison of the study and control groups by gender.

**Sex**	**Study group** **(*N* = 102)**		**Control group** **(*N* = 116)**		**In total**	
	**N**	**%**	**N**	**%**	**N**	**%**
**Women**	81	79.41	82	70.69	163	74.77
**Men**	21	20.59	34	29.31	55	25.23
**In total**	102	100	116	100	218	100

**Table 2:** Severity of depression symptoms in the study group according to the HDRS scale (*N* = 102).

**Variable**	**HDRS**	
	**N**	**%**
<7	0	0
8–12	13	12.74
13–17	22	21.57
18-29	64	62.75
>30	3	2.94
In total	102	100

**Notes.**

HDRS - Hamilton Depression Scale upon inclusion in the experiment - interpretation of results: <7 - no symptoms of depressive disorders; 8–12 - mild severity; 13–17 - moderate severity; 18–29 - severe severity; >30 - very severe intensification of depressive disorders symptoms.

**Table 3:** Analysis of the consistency of the genotype distribution of the GEMIN gene polymorphisms with the Hardy-Weinberg law.

**Polymorphism/gene**	***p*-** **value** ^*^		
	**Total**	**Control group**	**Study group**
rs3744741/GEMIN4	<0.0001	<0.0001	<0.0001
rs7813/GEMIN4	0.5	0.26	0.03
rs197388/GEMIN3	<0.0001	<0.0001	<0.0001

**Notes.**

* *p* < 0.05—in compliance with the Hardy-Weinberg equilibrium.

**Table 4:** Comparison of depression with the frequency of rs197388/GEMIN3 polymorphism genotypes.

**Model**	**Genotype**	**Control group**	**Study group**	**OR (95% CI)**	***p*-** **value** ^*^
Codominant	T/T	60 (51.7%)	47 (46.1%)	1.00	0.057
	A/T	29 (25%)	17 (16.7%)	0.75 (0.37–1.52)	
	A/A	27 (23.3%)	38 (37.2%)	1.80 (0.96–3.35)	
Dominant	T/T	60 (51.7%)	47 (46.1%)	1.00	0.41
	*A*/*T* − *A*/*A*	56 (48.3%)	55 (53.9%)	1.25 (0.74–2.14)	
Recessive	*T*/*T* − *A*/*T*	89 (76.7%)	64 (62.8%)	1.00	0.024
	A/A	27 (23.3%)	38 (37.2%)	1.96 (1.09–3.53)	
Overdominant	*T*/*T* − *A*/*A*	87 (75%)	85 (83.3%)	1.00	0.13
	A/T	29 (25%)	17 (16.7%)	0.60 (0.31–1.17)	

**Notes.**

* *p* < 0.05

Table 5 shows the distribution of the genotypes of the rs7813/GEMIN4 polymorphism compared to depression. A relationship of the G/A genotype in the codominant model, the A/A genotype in the recessive model, and G/A in the over-dominant model has been demonstrated. G/A genotypes in the codominant and over-dominant model (OR = 0.43; 0.35) reduce the risk of depression, while the AA genotype in the recessive model (OR = 2.66) increases this risk.

**Table 5:** Comparison of depression and the frequency of rs7813/GEMIN4 polymorphism genotypes.

**Model**	**Genotype**	**Control group**	**Study group**	**OR (95% CI)**	***p*-** **value** ^*^
Codominant	G/G	24 (20.7%)	30 (29.4%)	1.00	0.004
	G/A	75 (64.7%)	40 (32.2%)	0.43 (0.22–0.83)	
	A/A	17 (14.7%)	32 (31.4%)	1,51 (0.68–3.34)	
Dominant	G/G	24 (20.7%)	30 (29.4%)	1.00	0.14
	*G*/*A* − *A*/*A*	92 (79.3%)	72 (70.6%)	0.63 (0.34–1.16)	
Recessive	*G*/*G* − *G*/*A*	99 (85.3%)	70 (68.6%)	1.00	0.0031
	A/A	17 (14.7%)	32 (31.4%)	2.66 (1.37–5.17)	
Overdominant	*G*/*G* − *A*/*A*	41 (35.3%)	62 (60.8%)	1.00	0.004
	G/A	75 (64.7%)	40 (39.2%)	0.35 (0.20–0.41)	

**Notes.**

* *p* < 0.05

We also noticed (Table 6) that TT genotype of rs3744741/GEMIN4 polymorphism increased ORs twice in codominant as well as recessive model (OR = 2.01; *p* < 0.05 and OR = 2.23; *p* = 0.014 respectively). For all studied polymorphism the statistical power was around 0.8 (rs7813 −0.9071; rs197388 −0.7997; rs3744741 −0.7674). We have done a multivariate analysis adjusted for sex and age using SNPstats, however these factors did not change OR value more than 10%. We also found no relationship between studied SNPs and intensity of depression symptoms.

**Table 6:** Comparison of depression and the frequency of rs3744741/GEMIN4 polymorphism genotypes.

**Model**	**Genotype**	**Control group**	**Study group**	**OR (95% CI)**	***p*-** **value** ^*^
Codominant	C/C	74 (63.8%)	60 (58.8%)	1.00	0.02
	T/C	23 (19.8%)	11 (10.8%)	0.59 (0.27–1.31)	
	T/T	19 (16.4%)	31 (30.4%)	2.01 (1.03–3.91)	
Dominant	C/C	74 (63.8%)	60 (58.8%)	1.00	0.45
	*T*/*C* − *T*/*T*	42 (36.2%)	42 (41.2%)	1.23 (0.71–2.13)	
Recessive	*C*/*C* − *T*/*C*	97 (83.6%)	71 (69.6%)	1.00	0.014
	T/T	19 (16.4%)	31 (30.4%)	2.23 (1.17–4.26)	
Overdominant	*C*/*C* − *T*/*T*	93 (80.2%)	91 (89.2%)	1.00	0.063
	T/C	23 (19.8%)	11 (10.8%)	0.49 (0.23–1.06)	

**Notes.**

* *p* < 0.05

## Discussion

Genetic variations, such as single nucleotide polymorphisms (SNPs) in genes associated with miRNA maturation, can influence miRNA-dependent regulation of gene expression. In our own research, we looked for a relationship between GEMIN3 and GEMIN4 polymorphisms and the risk of depression. We have shown that in our population of patients, the risk of depression is almost doubled by polymorphic variants of the following genes: rs197388/GEMIN3 genotype A/A in the recessive model and rs3744741/GEMIN4 genotype T/T in the codominant and recessive model. The dual role of rs7813/GEMIN4 is notable, where the G/A genotype in the codominant and over-dominant model protects against depression. Our results for rs7813/GEMIN4 are different from the results mentioned results obtained by He et al. (2012), who reported that rs7813/GEMIN4 did not affect susceptibility to depression in their population of patients. The plausible reasons for these differences are as follows. The plausible reasons for these differences are as follows. First, First, He et al. (2012) calculated the ORs using a standard model with genotype and allele distributions. It could not be assumed that the recessive model in their analysis would have produced a result consistent with ours. Second, that research was conducted on an Asian population, while ours was conducted on a Caucasian population mingling with Central Europe. Therefore, the results may be different, as the incidence of depression depends not only on the genetic structure of the population but also on geographic and climatic factors (Daray et al., 2017; Arias-de la Torre et al., 2021). In addition, there are more severely and severely depressed people in our group, which may also explain this difference. Therefore, the SNPs GEMIN4 and GEMIN3 appear to substantially contribute to depression in the Polish population.

GEMIN3, also known as DDX20 or DP103, belongs to the DEAD-box RNA helicase family. Studies have shown that DEAD-box proteins play an important role in all aspects of cellular RNA metabolism (Curmi & Cauchi, 2018). DEAD-box proteins are generally attributed to specific processes, but growing evidence suggests that several of them are involved in more than one cellular process and sometimes play roles that are not directly related to RNA. GEMIN3 is known to be part of a large multiprotein complex, so called the survival motor neuron (SMN) (Liu et al., 1997; Charroux et al., 1999; Meister et al., 2001), which also includes the title protein SMN and seven different proteins, namely GEMIN2, GEMIN4-8, and Unrip (Cauchi, 2010). GEMIN4 does not directly bind SMN, but is transferred into the complex by interaction with GEMIN3 (Charroux et al., 2000; Otter et al., 2007). GEMIN4 is necessary for the formation of the Sm core *in vitro* (Shpargel & Matera, 2005), but little is known about the function of this protein. Research shows that GEMIN4 is an essential gene in mice. Mice lacking this gene die early in their embryonic development (Meier, Walker & Matera, 2018). Mourelatos et al. (2002) found that GEMIN3 and GEMIN4 proteins are present in the 15S ribonucleoprotein complex that contains eIF2C, which is crucial for miRNA processing. The researchers conclude that GEMIN4 plays a pivotal role in the biogenesis of mammalian ribonucleoproteins and that this protein has a dominant effect on the localization of SMN, GEMIN3 and other members of the SMN complex. This relocation effect is entirely dependent on the presence of eight NLS amino acids in the N-terminal region of GEMIN4 (Meier, Walker & Matera, 2018). Does GEMIN4 change the location of its dependent relationships depending on the genotype and thus play a dualistic role, *i.e.*, protects or contributes to depression?

GEMIN4 is also included in the group of corepressors included in coregulators. Coregulators, composed of coactivators and corepressors, are cellular factors that interact with nuclear receptors to enhance or attenuate transactivation (McKenna & O’Malley, 2000). Unlike nuclear receptors, coregulators are structurally and functionally differentiated and are often recruited in a ligand- and type-specific manner. They have been found to play a key role in modulating gene expression through nuclear receptors and are believed to confer tissue specificity and ligand activity on the receptor (Kodera et al., 2000; Kraichely et al., 2000; McKenna & O’Malley, 2000; Klokk et al., 2007). GEMIN4 turned out to be a corepressor of steroid hormone receptors. It represses the mine ralocorticoid receptor, suppresses transcription mediated by the glucorticosteroid-GR receptor; adrenocorticotropic-AR and progesterone-PR (Yang et al., 2015). Other researchers report that GEMIN4 itself would not have had such an effect if it were not for its association with GEMIN3. This close association of GEMIN4 with GEMIN3 suggests that it may act as a cofactor of putative ATPase activity and/or GEMIN3 helicase. The role of GEMIN3 as a transcription regulator has been confirmed by reports describing the ability of its nonconservative C-terminal domain to interact with and modulate various cell transcription factors. GEMIN3 has been found to suppress steroidogenic factor 1 (SF-1) (Ou et al., 2001; Yan et al., 2003) and growth response protein 2 (Egr2/Krox-20) (Gillian & Svaren, 2004).

Steroidogenic factor 1 (SF-1) is an orphan nuclear receptor protein. It binds to a DNA sequence called the hormone response element (HRE) and regulates the transcription of target genes. SF-1 regulates the expression of genes involved in sex differentiation, control of pituitary gonadotropin secretion, and the synthesis of steroid hormones in the adrenal cortex and gonads (El-Khairi & Achermann, 2012). It should be noted that in patients with depressive syndromes, especially of the endogenous type, disorders of the pituitary, adrenal cortex, and other endocrine glands are quite often marked. It should be emphasized that hypercortisolemia and altered rhythm of secretion of this hormone persisted in a significant number of depressed patients, while about 50% of patients did not demonstrate inhibition of its secretion after dexamethasone administration (Gallagher, Reid & Ferrier, 2009).

Does the GEMIN4 polymorphism increase the risk of depression by weaker suppression of steroidogenic factor 1 and therefore increase the activity of the HPA axis in carriers of the AA genotype? The opposite might be true, that is, the protective function of GEMIN4 for GA genotype carriers involves an increased suppression of steroidogenic factor 1 by GEMIN3. Perhaps future studies will provide an answer to this question. The GEMIN polymorphism may be associated with growth response proteins 2 (Krox20/EGR2). Krox20/EGR2 is one of four growth response genes. Early growth response genes (EGR), including EGR1, EGR2, EGR3, and EGR4, encode DNA binding transcription factors that contain zinc fingers (Gabet et al., 2010). EGR genes encode a family of direct early transcription factors that mediate the transcription of various genes related to neuronal development and plasticity, cognition, circadian rhythm, and social behavior (Gabet et al., 2010). EGRs are involved in the pathophysiology of schizophrenia and bipolar disorder, as well as the mechanisms of action of antipsychotic drugs. EGR2 plays an important role in peripheral nerve myelination and T cell maturation, hindbrain segmentation, and lipid biosynthesis (Topilko et al., 1994; Zorick et al., 1999; Parkinson et al., 2004).

The involvement of EGR2 in cognition has also been reported. In Korea, a link has been found between EGR2 and bipolar disorder. The authors suggested that EGR2 may be a susceptibility gene for this disease (Kim et al., 2012). Deletion of Krox20 in mice is associated with a reduced ability to code for the Krox20 gene protein (including the zinc finger DNA binding domain). These mice do not survive postnatally and show severe hindbrain defects (Wilkinson et al., 1989; Swiatek & Gridley, 1993).

The relationship between an occurrence of depression and the polymorphisms in GEMIN3 and GEMIN4 that we analyzed can also be explained by the mechanisms of involvement of these genes in the development of neoplasms. GEMIN3 appears to be involved in the activation of I*κ*B 2 (IKK2) kinase. IKK2 kinase is key to activation of the NF-*κ*B transcription factor that acts on metalloproteinase 9. Increased expression of active NF-*κ*B (phospho-p65) and metalloproteinase 9 (MMP9) forms the GEMIN3—NF-*κ*B—MMP9 axis in breast cancer metastases.

Metalloproteinases degrade the extracellular matrix. This process occurs and is essential in tumor metastasis. The role of MMPs in the function of the blood–brain barrier and stability of the myelin sheaths is also noted. Participation of MMPs in the mechanisms of synaptic plasticity is also important. MMP-9 has the greatest ability to degrade type IV collagen, which is the main component of the basal membrane. Mateloproteinases in the excitatory synapse play a role in memory, learning, and synaptic plasticity (Pantazopoulos et al., 2021), *i.e.*, the ability of nerve cells to permanently change under the influence of an external stimulus. Recent reports indicate that MMP-9 mediates inflammation after oxidative stress in the central nervous system (CNS). Oxidative stress in adolescence contributes to the development of inflammation in the CNS, disturbed the development of the neural network, and myelination (Chelini et al., 2018). The role of oxidative stress in the development of both schizophrenia and affective diseases has been described (Lindqvist et al., 2017), which is another argument confirming that MMP-9 is important in the pathogenesis of these diseases and GEMIN genes through MMP9 and inflammation that is possibly associated with depression. Inflammation is a known etiological factor in depression. The role of GEMIN in tumor development through the GEMIN3—NF-*κ*B—MMP9 axis is complicated by the fact that the role of GEMIN3 in carcinogenesis is different for different types of cancer. In contrast to its role as a tumor promoter in breast, prostate, and colon cancer, GEMIN3 acts as a tumor suppressor in hepatocellular carcinoma. GEMIN3 has been found to reduce NF-*κ*B activity by regulating the inhibitory function of NF-*κ*B miRNA-140 in liver cancer progression (Takata et al., 2012; Takata et al., 2013).

At the moment, we cannot clearly identify the mechanisms linking the GEMIN polymorphism with depression. We still do not fully understood precise mechanisms through which GEMIN works in depression. However, since the discovery of GEMIN, which happened over two decades ago, impressive progress has been made, its function in selected physiological and pathological processes has been described. We wonder to what extent the research will help us to understand the role of the key RNA DEAD-box helicase in cellular metabolism. Some intrinsic limitations should be considered. First, all participants are limited to the European Caucasian population, therefore, whether our finding are applicable to other populations requires further research to confirm. Second, this is a hospital-based, single-center study, so the selection bias cannot be excluded.

The genotype distribution of some of the polymorphisms we analyzed (rs197388 and rs3744741) was inconsistent with HWE condition. The HWE is a fundamental principle of population genetics, according to which population genotype frequencies, if not externally disturbed, are constant throughout generations. There are several reasons for the HWE imbalance. Errors in genotyping, in particular those occurring in case-control association studies, are commonly suggested. This assumption is based on the belief that in a large, randomly mating population, the frequency of genotypes should be consistent with the HWE equilibrium. However, alleged genotyping errors cannot be grounds for an unequivocal rejection of study results. First, genotyping errors are generally too few to deviate from the HWE. Moreover, the number of observed heterozygotes is an important parameter, suggesting a significant contribution of genotyping errors to deviation from the HWE. The decrease in the frequency of heterozygosity (LoH) is indicative of factors other than genotyping errors responsible for deviations from the equilibrium of HW. Factors include clean-up selection, copy number variation, inbreeding, and population substructure. An additional analysis verifying the correctness of genotyping is recommended if an excess of heterozygotes (GoH) is observed. The analysis of the frequency of heterozygosity performed in our study does not indicate an occurrence of the GoH phenomenon and thus no errors in genotyping. Of note, Hardy-Weinberg disequilibrium that is caused by most interesting biologic phenomena typically results in excess homozygosity, as observed in our study. However, most probably violation of Hardy-Weinberg equilibrium in our study population implies a selected rather than a random sample as more women are affected depression than man in Poland. This is not surprising as he risk of depressive disorders among women is about twice as high as among men (Kessler, 2003; Munce & Stewart, 2007). There are several hypotheses regarding the reasons for this phenomenon. First, accepted social norms in our culture justify women’s expression of depressive feelings (Newman et al., 2006). In addition, learned helplessness is more common among women (Silverstein & Lynch, 1998). According to Kendler, Thornton & Prescott (2001) women are also more likely to experience unpleasant events during their lives and are more likely to react with mood disorders in response. Hormonal fluctuations in the monthly cycle and over the course of a woman’s life may also contribute to this group’s greater susceptibility to depression (Kendler et al., 2006). There are also theories that women who carry the depression gene become depressed, while men who carry the same gene become alcohol dependent (Wenzel, Steer & Beck, 2005). Therefore, we had to fit control group to the case group. A sample size may affect the precision of the results. Regarding our study, a relatively small size was the greatest methodological limitation. Our findings should be confirmed in future larger-scale study, specifically designed and adequately powered to detect genotype-specific differences in patients with depression. Third, the mechanism of studied polymorphisms on the occurrence of depression is still unclear, and the analysis of biological functions is needed to further research. The possible explanation is that SNP loci is located in the exon, the functional region of the GEMIN4 and GEMIN3 genes, which may play a potential role in the regulation of protein expression. The expression of the GEMIN4 protein is tightly associated with the biogenesis of related miRNAs, which may alter the risk of depression. Despite the above limitations, our research results provide scientific evidence for the impact of GEMIN4 genetic variability on the risk of depression in future studies.

## Conclusions

We believe that SNPs GEMIN4 (rs7813 and rs3744741) and GEMIN3 (rs197388) are associated with a risk of depression. However, it is advisable to confirm our observations on a larger group of depression patients to provide more reliable evidence.

##  Supplemental Information

10.7717/peerj.14317/supp-1 Supplemental Information 1 Results Codebook: 0—control; 1—depression

## Additional Information and Declarations

### Data Availability

The following information was supplied regarding data availability: The raw data is available in Tables 1–4.
